# Life Course Trajectories for Young Pasifika in Aotearoa: Protocol for the 25-Year Follow-Up of the Pacific Islands Families Study Cohort

**DOI:** 10.2196/77460

**Published:** 2025-11-10

**Authors:** El-Shadan Tautolo, Tugce Bakir-Demir, Shabnam Jalili-Moghaddam, Faasisila Savila, Jesse Kokaua, Philip J Schluter, Maulupeivao Betty Ofe-Grant, Elaine Rush, Jalal Mohammed, Sione Vaka, Jemaima Tiatia-Siau, Radilaite Cammock, Braden Te Ao, Jacinta Fa'alili-Fidow, 'Ilaisaane M E Fifita, Sam Manuela, Leon Iusitini

**Affiliations:** 1 Faculty of Health and Environmental Sciences, Auckland University of Technology, AUT South Campus, MB Building, 640 Great South Road, Auckland, 2202, New Zealand, 64 9219999, ext 7527; 2 Faculty of Medical and Health Sciences, University of Auckland, Auckland, New Zealand; 3 Centre for Pacific Health, University of Otago, Dunedin, New Zealand; 4 Faculty of Health, University of Canterbury, Christchurch, New Zealand; 5 Faculty of Health, The University of Queensland, Brisbane, Australia; 6 Faculty of Business, Economics and Law, Auckland University of Technology, Auckland, New Zealand; 7 Centre of Research Excellence, Riddet Institute, Palmerston North, New Zealand; 8 Umanand Prasad School of Medicine & Health Sciences, University of Fiji, Lautoka, Fiji; 9 Division of Health, University of Waikato, Hamilton, New Zealand; 10 Faculty of Arts and Education, University of Auckland, Auckland, New Zealand; 11 Moana Connect, Auckland, New Zealand; 12 Faculty of Science, University of Auckland, Auckland, New Zealand

**Keywords:** pacific youth health, longitudinal research, life course epidemiology, determinants of health, psychological wellbeing, nutritional and metabolic wellbeing, economic wellbeing, protocol

## Abstract

**Background:**

From birth, many young Pacific people in Aotearoa New Zealand experience a disproportionately high burden of psychological distress, metabolic disease, and socioeconomic disparities within education and employment which contribute to significant health inequalities. Further research is needed to understand the drivers influencing these outcomes.

**Objective:**

This paper provides a comprehensive overview of the quantitative component of the Pacific Island Families Study: Ala mo Tupulaga Pasifika Aotearoa (PIF:ATP; Life Course Trajectories for Young Pasifika in Aotearoa), the latest follow-up of the longitudinal PIF birth cohort study, which uses a mixed-methods approach.

**Methods:**

The PIF Study is a multidisciplinary longitudinal study that tracks the health and development of 1398 Pacific children born in 2000 at Middlemore Hospital, South Auckland, Aotearoa, New Zealand. Data collection has occurred at 10 time points from infancy through young adulthood, with this PIF:ATP assessment phase occurring at ages 25‐26 years, which aims to reach at least 750 cohort members. The assessments will take place at participants’ homes or at Auckland University of Technology for those residing in Auckland. Data collection will be conducted across multiple sites, including Auckland, Wellington, Hamilton, and Whangārei in Aotearoa New Zealand, as well as Brisbane, Sydney, and Melbourne in Australia. Physical measurements such as weight, height, waist and hip circumferences, grip strength, body fat mass and muscle mass, blood pressure and pulse, glucose and lipid screening, and skin carotenoid concentration will be undertaken. In addition, self-reported data will be collected on psychological well-being (eg, depression, anxiety, and family functioning), nutritional and metabolic well-being (eg, food intake and physical activity), and economic well-being (eg, educational attainment, employment status, and job occupation and industry).

**Results:**

Data collection is scheduled to commence in June 2025 and conclude by December 2026. The first set of results and analysis is expected to be published from December 2027 onward. Reporting of all results will comply with the Strengthening the Reporting of Observational studies in Epidemiology (STROBE) guidelines.

**Conclusions:**

This paper presents the protocol for the 25-year follow-up of the first Pacific longitudinal cohort study, which will comprehensively examine psychological, nutritional, metabolic, and economic well-being of Pacific young adults. With 25 years of longitudinal data and extensive expertise in life course research, this protocol outlines the design, methodology, and scope of the quantitative component of the PIF:ATP research program. This phase is uniquely positioned to address key issues identified by Pacific communities and generate evidence to inform meaningful interventions and guide policy development while providing robust, contemporary, high-quality empirical evidence.

## Introduction

### Background

Pacific peoples in Aotearoa, New Zealand represent a diverse mix of ethnicities and cultural groups, including migrants and their descendants from Samoa, Tonga, the Cook Islands, Fiji, Niue, Tokelau, Tuvalu, and other Pacific nations across Polynesia, Melanesia, and Micronesia. Census data reveal that 8.9% of New Zealand’s population identify as being of Pacific ethnicity [1], with two-thirds of them born in New Zealand [2]. This underscores a demographic shift, with the majority of Pacific peoples now being a local rather than an immigrant population.

The New Zealand Pacific population is youthful, with more than half (58.7%) aged 29 years or younger, in contrast to the New Zealand European population, where a smaller proportion (35.9%) falls within this age range [1]. However, they experience a disproportionately high burden of psychological distress and increased risk of developing poor metabolic outcomes compared to the general population [3-8]. Additionally, they face significant disparities in tertiary education and employment, which often contribute to long-term health inequalities [9-11]. While existing evidence highlights a complex interplay of cultural, familial, and economic factors that influence health and well-being, there remains a critical gap in understanding the specific mechanisms underlying these influences and their relevance to Pacific young adults. Building on 25 years of research investment, the Pacific Island Families Study: Ala mo Tupulaga Pasifika Aotearoa (PIF:ATP) research program, which translates as “Life Course Trajectories for Young Pasifika in Aotearoa,” is an epidemiologically robust study designed to explore these mechanisms as Pacific young adults navigate critical life course transitions.

The PIF Study is a multidisciplinary longitudinal investigation of the health and development of Pacific peoples in New Zealand. Beginning in 2000, the study follows a birth cohort of 1398 Pacific children born at Middlemore Hospital in South Auckland, representing one of the largest and longest-running longitudinal studies of Pacific health globally [12,13]. Data have been collected across 10 waves to date, with assessments at 6 weeks, 1 year, 2 years, 4 years, 6 years, 9 years, 11 years, 14 years, 17 years, and 22 years post partum (Table 1). The PIF Study embodies a Pacific-centered approach, initially advocated by Pacific communities, led by Pacific researchers, and dedicated to advancing Pacific health and development. This reflects a commitment to community-driven research while promoting Pacific leadership in academia.

**Table 1.** An overview of cohort follow-up and retention across measurement waves.

Informant type	6 weeks	1 year	2 years	4 years	6 years	9 years	11 years	14 years	17 years	22 years
Teacher questionnaire	—^a^	—	—	—	559	692	707	—	—	—
Child assessment	—	—	1064	909	897	891	952	931	632	471^b^
Paternal interview	—	825	757	—	591	790	646	—	—	—
Maternal interview	1376	1224	1144	1048	1001	996	1029	958	—	—

a Not available.

b The participant numbers were lower than anticipated due to significant disruptions during the data collection phase. This period coincided with Severe Tropical Cyclone Gabrielle and the Auckland Floods, which led to a national state of emergency being declared on February 14, 2023 [14].

The PIF:ATP extension now examines these participants at ages 25‐26 years, focusing on their transition into adulthood. It uses mixed methods and multifaceted approaches, combining quantitative and qualitative data collection to capture the complexity of health and development within the Pacific context. This protocol paper only covers the quantitative element of the study. As in previous phases, qualitative themes will be guided by preliminary descriptive quantitative analyses which will identify potential areas of inquiry. These areas can then be explored in depth using qualitative methods, providing further insight into the underlying drivers of patterns revealed by the survey.

Following the quantitative and qualitative phases, findings will be translated into practice through collaboration with Pacific health providers, policymakers, and key stakeholders. This will ensure research outcomes inform service delivery, policy decisions, and health promotion activities for Pacific communities. While this article focuses on the quantitative aspects of the study, the overarching themes—psychological well-being, nutritional and metabolic well-being, and economic well-being—will be explored across the study’s quantitative, qualitative, and knowledge translation and implementation phases.

### Psychological Well-Being

Pacific peoples report high levels of life satisfaction, family well-being, and strong social connections and support from family and friends [3,15]. However, they also experience significant health and socioeconomic disparities compared to other New Zealanders. In particular, mental health inequalities are pronounced, with Pacific peoples experiencing higher rates of psychological distress, mental disorders, and suicidal behaviors [3-7,16]. These disparities represent a critical public health concern in New Zealand.

Young Pacific people face the greatest challenges, reporting the highest levels of mental distress, self-harm, and suicide mortality among all Pacific age groups [3,17-19]. Alarmingly, these disparities emerge early in life. Data from the New Zealand Health Survey show that Pacific children are more likely than their non-Pacific peers to experience emotional difficulties and behavioral problems, even after adjusting for age and sex [20]. Similarly, the Growing Up in New Zealand study—a longitudinal birth cohort study that began in 2009‐2010, involving approximately 6000 children and their families—found that, at the age of 8 years, Pacific children reported higher levels of depressive and anxiety symptoms compared to European and Asian children [21]. This disproportionate burden on young Pasifika is part of a broader “pandemic of psychological distress among youth” in New Zealand [22].

Cultural identity, sense of belonging, and engagement in cultural practices [23-26] are important drivers of psychological well-being among Pacific peoples. However, some inconsistent findings warrant further investigation. For instance, mothers who maintained strong connections to their Pacific culture but a relatively weak connection to mainstream New Zealand culture (Separators) or who engage with both Pacific and New Zealand culture (Integrators) exhibited elevated depressive symptoms relative to those who either adopted the mainstream New Zealand culture and relinquished their Pacific culture (Assimilators) or were culturally disengaged (Marginalists) [27]. Interestingly, another PIF Study analysis revealed contrasting patterns: in unadjusted analyses, Marginalists showed significantly greater odds of mental disorder symptoms than Integrators, while Separators demonstrated significantly lower odds. However, after statistical adjustment, these odds were nonsignificant, suggesting confounding [25]. Nonetheless, these discrepancies highlight the need for additional studies to better understand the complex relationships between cultural identity and mental health outcomes in Pacific populations.

Family functioning, such as parent-child relationships, parenthood, and intergenerational cultural differences, also plays a critical role in shaping mental health outcomes. Findings from the PIF Study reveal that Pacific children who report strong relationships with their parents, and whose parents demonstrate high levels of positive parenting, experience significantly lower rates of depressive symptoms [26,28]. However, findings from the New Zealand Mental Health Monitor and Health and Lifestyles Survey underscore that stigma surrounding mental health remains a significant issue within Pacific communities [3]. Qualitative research with Tongan youth revealed concerns that traditional interpretations of mental distress, commonly held by family and community members, often perpetuate shame and stigma [29]. Despite these insights, the interplay between individual, familial, societal, and structural determinants of Pacific mental health remains poorly understood. Further research is needed to disentangle these factors and better inform culturally responsive interventions [22].

### Nutritional and Metabolic Well-Being

Nutrition-related noncommunicable diseases (NCDs), such as obesity, type 2 diabetes, and heart disease, are major health concerns for Pacific communities in New Zealand and across the Pacific region [30,31]. Findings from the PIF Study show that the cohort has experienced rapid weight gain, with more than 70% classified as overweight or obese by the age of 14 years [8]. Many also exhibit early signs of metabolic risk, increasing their likelihood of developing NCDs later in life [32]. As an example, there is increasing evidence suggesting that elevated serum uric acid in children and adolescents is a risk factor for type 2 diabetes, hyperuricemia, gout [33,34], cardiometabolic diseases [35], and metabolic syndrome [36] later in life. Results from a nested study of a subsample of PIF Study cohort members (n=204) at the age of 14‐15 years showed that these children have high serum uric acid levels with a positive association between appendicular skeletal muscle mass and serum uric acid in both sexes [37].

Childhood physical growth and weight gain are shaped by a complex interplay of factors, including parental perceptions, cultural influences, gender, acculturation, and environmental conditions [38,39]. For example, nutrition and metabolic health outcomes among Pacific youth can be moderated by family and cultural factors, such as the positive impact of parental education on children’s BMI [40] and the negative impacts of food insecurity on body composition [41] and educational achievement [42]. However, how these childhood determinants translate into adult health outcomes remains unclear.

As the PIF Study cohort transitions into adulthood, they continue to face significant challenges related to food intake and metabolic health. Earlier findings from the PIF Study found food purchasing decisions of mothers are driven more by expiry dates, cost, and perceived palatability rather than by food labeling or nutritional knowledge [43]. At the age of 14‐15 years, the majority consumed sugary drinks more than twice daily, and 75% reported consuming sugar-containing foods at least 4 times per day [37]. A better understanding of how Pacific youth navigate food literacy and food security, particularly amid rising living costs, is essential. These findings suggest that existing public health messaging may not be effective in influencing healthier food choices among Pacific youth.

While food intake and eating behaviors play a crucial role in shaping long-term health outcomes, physical activity and sedentary behaviors during childhood and adolescence also significantly influence health trajectories. A systematic review and meta-analysis of longitudinal studies showed a prospective negative association between muscular fitness in childhood and adolescence and levels of adiposity and cardiometabolic parameters in later life, alongside a positive association with bone health [44]. Pacific peoples are considered among the most muscular populations globally. Compared to other ethnic groups, Pacific adults [45] and children [46] tend to have greater muscle mass and less body fat relative to their height and weight. Traditionally, they also possess a larger body frame and a higher proportion of central fat compared to peripheral fat, traits that may reflect adaptations developed before and during their migration across the Pacific Ocean [47].

Building on this understanding, findings from the PIF Study further illustrate the role of body composition in physical performance. Among the nested subsample at the age 14‐15 years, a 6-minute walk test was conducted, with heart rate measurements recorded at 0, 6, and 7 minutes. Results showed that after controlling for age, each kilogram increase in body weight was associated with a shorter walking distance for boys, but no change in walking distance for girls. Heart rate change during the walk test increased in both groups [48]. These results highlight how body composition differences can impact physical performance among Pacific youth.

Despite young adulthood being a critical period for shaping lifelong health trajectories [49], research on Pacific young adults’ nutrition, metabolic health, and physical activity remains limited. Furthermore, recognizing young adults as a distinct subpopulation in health research and policy [50] could provide key insights into the long-term determinants of NCDs and inform more effective, culturally responsive strategies for prevention and intervention.

### Economic Well-Being

In addition to the aforementioned psychological, nutritional, and metabolic challenges, economic well-being plays a crucial role in the overall well-being of Pacific populations in New Zealand. Despite enrolling in tertiary education at similar rates to the general population, Pacific peoples face significant barriers to academic success and career advancement [51]. This is evident in their underrepresentation in higher levels of education, such as bachelor’s degrees, and lower tertiary course completion rates which were 12% points lower than the New Zealand average in 2022 [52-54]. In the workforce, Pacific peoples experience higher rates of unemployment and not in employment, education, or training than the general population [55]. Recent studies indicate that Pacific workers are disproportionately concentrated in the manufacturing, construction, and retail and hospitality sectors, predominantly employed as laborers, plant and machinery operators, and customer service representatives. This occupational clustering reinforces labor market segregation and systemic inequalities, with limited opportunities for upward career mobility [56,57]. In contrast, Pacific peoples hold only 5.1% of senior managerial and leadership positions, primarily within the public sector [58].

These educational and labor market disparities contribute to lower average income and wealth among Pacific peoples [59-61]. The “Pacific pay gap,” where Pacific workers, particularly Pacific women, earn lower wages than other ethnic groups, has been attributed in part to systemic racism, unconscious bias, and discriminatory workplace practices [57]. This entrenched inequity reflects broader structural inequalities and limits the economic advancement and social mobility of Pacific communities in New Zealand. These disparities also have intergenerational consequences, as differences in parents’ education and socioeconomic status account for gaps between European and Pacific peoples in tertiary enrollment and achievement [62]. Thus, educational and socioeconomic inequalities among the current generation of young Pacific peoples will have sequelae for the next generation.

The impact of these economic disparities extends beyond economic struggles, influencing health outcomes significantly. As noted in the Ministry of Health’s Te Mana Ola: Pacific Health Strategy in New Zealand*,* “Pacific peoples experience longstanding and unacceptable health inequities…[which]…are strongly linked to the wider determinants of health such as education, employment, and housing” [63]. These inequities are evident in rates of psychological distress, which follow a socioeconomic gradient. Adults with the lowest material living standards experience distress rates 30 times higher than those with the highest living standards [9]. A similar gradient is also observed for Pacific self-harm and suicide rates across levels of neighborhood deprivation [6,18,19]. Additionally, BMI and waist circumference among the total population, and dietary behaviors of adolescents, are strongly associated with socioeconomic status in New Zealand [10,11]. Thus, addressing the socioeconomic disparities experienced by Pacific peoples is critical not only in their own right but also due to their spillover effects on health outcomes. While research on these issues remains limited, it is evident that the complex factors contributing to these disparities accumulate across the life course, beginning from gestation and continuing into adulthood. Longitudinal and cross-sectional data from PIF:ATP are essential for understanding why young Pacific peoples face educational and labor market disparities in New Zealand. These data can identify critical intervention points across the life course and inform the development of more effective services in education, employment, and social welfare to promote equitable outcomes for Pacific young adults.

The 3 themes of PIF:ATP—psychological, nutritional and metabolic, and economic well-being—are theoretically underpinned by the key features of the Fonofale model of Pacific health and well-being [64]. The Fonofale model reflects the interconnected nature of well-being from a Pacific perspective, emphasizing a holistic view where physical, mental, and social health are interdependent. Well-being is maintained when these dimensions are in balance and harmony. This holistic health is imbedded in family (the foundation of the fale or house) and culture (the roof of the fale), with well-being domains depicted as structural pillars of the fale. Within this framework, the concept of self is relational: an individual’s sense of identity is deeply connected to family and culture, and any disconnection from these relationships can disrupt their overall well-being [64]. Such disconnections can arise from an inability to meet social obligations to family, village, and church (via fa’alavelave or gifting, remittances, and tithing). In short, connections to family and culture, and having the economic means to meet familial and cultural commitments, are central to Pacific views of well-being [65]. The 3 studies within the PIF:ATP program therefore capture the critical features of Pacific health and well-being as conceptualized in the Fonofale model.

### Study Objectives

The quantitative component of the PIF:ATP program has three objectives: (1) quantitatively measure outcomes in psychological well-being, nutritional and metabolic well-being, and economic well-being among 25-year-old Pacific young adults; (2) consider the interrelations between these outcomes (eg, how mental distress is related to dietary patterns and employment status, how nutrition is related to occupation and mental health); and (3) identify risk and protective factors related to each set of well-being outcomes.

Specifically, we will examine sociocultural, environmental, and employment factors that influence the psychological well-being of Pacific young adults in either positive ways (eg, increased resilience to mental distress or protection of psychological well-being) or negative ways (eg, increased vulnerability to mental distress), and the mechanisms through which these factors operate. For nutritional and metabolic well-being, we will use comprehensive physiological measurements including anthropometrics (height, weight, BMI, waist, and hip circumference), body composition analysis (fat mass and muscle mass), blood pressure, metabolic screening (nonfasting glucose and lipid profiles), functional assessments (grip strength), and biomarker analysis (skin carotenoids). These measures will allow us to identify factors contributing to both favorable metabolic outcomes (such as healthy lipid profiles and normotensive status) and adverse outcomes (including obesity-related parameters and diabetes risk indicators). In addition, physical activity levels and sedentary behaviors will be assessed. The economic well-being component will examine educational and employment trajectories, with particular attention to factors that facilitate tertiary course completion, stable employment, and career progression, as well as those that lead to educational discontinuation, unemployment, or precarious employment. Throughout all analyses, we will pay particular attention to the underlying mechanisms connecting these various dimensions of well-being among Pacific young adults.

## Methods

### Study Design and Population

The PIF:ATP program will follow up the PIF Study birth cohort at the age of 25 years. Assessments and interviews with the PIF Study cohort have been conducted at multiple developmental stages, including at 6 weeks and 1 year, 2 years, 4 years, 6 years, 9 years, 11 years, 14 years, 17 years, and 22 years postpartum (see Table 1 for an overview of e-cohort follow-up numbers across measurement waves) [8,13]. The upcoming wave of data collection, scheduled for 2025‐2026, will target participants aged 25-26 years and aims to engage at least 750 cohort members. Many cohort members have left New Zealand and moved overseas since birth, particularly to Australia, and there has been much internal migration within New Zealand as well. There are no exclusion criteria for participation in the PIF:ATP Study. However, of the original cohort, 138 participants have formally withdrawn, and 7 are deceased. The assessments will take place at participants’ homes or at Auckland University of Technology (AUT) for those residing in Auckland. Data collection will be conducted across multiple sites, including Auckland, Wellington, Hamilton, and Whangārei in New Zealand, as well as Brisbane, Sydney, and Melbourne in Australia. While most participants currently reside in Auckland, 23% have relocated to Australia, and 10% live elsewhere in NZ, primarily in Wellington, Hamilton, and Whangārei. This phase will focus on collecting cross-sectional data on psychological, nutritional and metabolic health, and economic well-being, while also incorporating relevant longitudinal data from earlier waves into analyses where appropriate.

### Study Recruitment and Procedures

A total of 6 field researchers will first attempt to contact participants via phone using the contact details held by the PIF Study. If a participant’s phone number has changed or is no longer in use, researchers will follow up via email or a home visit. If these efforts are unsuccessful, they will reach out to the participant’s nominated relatives (eg, mothers, fathers, and 2 additional contacts provided in previous study phases) to help re-establish contact. In addition, flyers will be distributed within Pacific communities in Auckland to increase outreach to original cohort members. In cases where direct contact methods are unsuccessful, participants’ publicly available social media accounts will also be used to reach re-establish contact.

During the initial phone call, field researchers will provide potential participants with detailed information about the 25-year follow-up, including what their participation entails. Researchers will also address any questions the participant may have before scheduling an interview appointment. Participants will be advised to wear loose-fitting clothing to accommodate a blood pressure cuff. They will also be informed that a small blood sample will be collected via finger-prick test. Additionally, they will be asked to avoid food, alcohol, caffeine, cigarettes, and exercise for at least 30 minutes before the appointment, as these factors may temporarily impact some of the physical measurements such as elevating blood pressure.

At the in-person visit, conducted at the participant’s home or at AUT campuses, the field researcher will provide a detailed explanation of all study procedures, including physical measurements. Participants will complete questionnaires related to psychological well-being while the researcher prepares equipment for the physical assessments. Once the survey is completed, physical measurements will be taken, followed by completion of additional questionnaires covering food intake, physical activity, and economic well-being. The entire assessment is expected to last approximately 2 to 2.5 hours.

### Outcomes

#### Physical Measurements

Please see Table 2 for an overview of the physical measurements.

**Table 2.** Overview of physical measurements to be taken in Pacific Islands Families Study: Ala mo Tupulaga Pasifika quantitative study (25-year follow-up).

Variable	Instrument	Supplier	Unit precision	Precision of measurement	Final value	Type
Height	SECA stadiometer	Seca GmbH and Co KG, Hamburg	0.1 cm	0.5 cm	Average of 3 valid measurements	Continuous
Weight	Patient weight scale	Wedderburn scales Ltd	0.1kg	0.5 kg	Average of 3 valid measurements	Continuous
BMI	Derived (weight/height²)	—^a^	0.1 kg/m²	—	Calculated once	Continuous
Waist circumference	SECA anthropometric measuring tape	Seca GmbH and Co KG, Hamburg	0.1 cm	0.5 cm	Average of 3 valid measurements	Continuous
Hip circumference	SECA anthropometric measuring tape	Seca GmbH and Co KG, Hamburg	0.1 cm	0.5 cm	Average of 3 valid measurements	Continuous
Waist-to-hip ratio	Derived	—	0.1	—	Calculated once	Continuous
Waist-to-height ratio	Derived	—	0.1	—	Calculated once	Continuous
Body fat mass	Tanita BC-545N	Tanita Corporation, Tokyo	0.10%	—	Average of 3 valid measurements	Continuous
Muscle mass	Tanita BC-545N	Tanita Corporation, Tokyo	0.1kg	—	Average of 3 valid measurements	Continuous
Blood pressure	Omron HEM7121	Omron Healthcare, Kyoto	1 mm Hg	10 mm Hg	Average of the last 2 valid measurements	Continuous
Pulse rate	Omron HEM7121	Omron Healthcare, Kyoto	1 bpm	10 bpm	Average of the last 2 valid measurements	Continuous
Blood glucose	CardioChek PA Analyzer	PTS Diagnostics	0.1 mmol/L	—	Single measurement	Continuous
Total cholesterol	CardioChek PA Analyzer	PTS Diagnostics	0.1 mmol/L	—	Single measurement	Continuous
HDL^b^ cholesterol	CardioChek PA Analyzer	PTS Diagnostics	0.1 mmol/L	—	Single measurement	Continuous
Grip strength	Camry-EH 101-Digital Hand Dynamometer	Camry	0.01 kg	—	Maximum of 3 measurements	Continuous
Skin carotenoids	Veggie Meter	Longevity Link, Utah	1 Carotenoid Score	—	Average of 3 valid measurements	Continuous

a Not applicable.

b HDL: high-density lipoprotein.

##### Height

Height (cm) will be measured using a portable SECA stadiometer (Seca GmbH and Co) without shoes. A total of 3 measurements will be taken, with a maximum allowable difference of 0.5 cm. If the difference exceeds this threshold, additional measures will be taken until consistency is achieved. The final recorded height will be the average of the 3 valid measurements.

##### Weight

Weight (kg) will be measured using patient weight scale (Wedderburn-WMMS6111) with participants barefoot and wearing light clothing. A total of 3 weight measurements will be recorded, with a maximum allowable difference of 0.5 kg. If the variation exceeds this threshold, additional measures will be taken until consistency is reached. The final weight will be the mean of the 3 valid measurements.

##### BMI

BMI will be calculated using the standard formula: BMI=weight (kg)\height^2^ (m). This measure provides an indicator of body weight relative to height.

##### Waist Circumference

Waist circumference (cm) will be measured using a SECA anthropometric nonstretchable measuring tape at the midpoint between the lower margin of the last palpable rib and the top of the iliac crest. Participants will stand in a relaxed posture, with arms at their sides and feet together. A total of 3 measurements will be taken, ensuring a maximum difference of 0.5 cm. If exceeded, additional readings will be taken. The final recorded value will be the average of the 3 valid measurements.

##### Hip Circumference

Hip circumference (cm) will be measured using a SECA anthropometric measuring tape at the level of the greatest buttock protrusion, ensuring that the tape remains parallel to the floor and snug but not compressing the skin. Participants will stand with their feet together and weight evenly distributed, and any bulky clothing will be removed to ensure accuracy. A total of 3 measurements will be taken, with a maximum allowable variance of 0.5 cm. If exceeded, measurements will be repeated. The final value will be the average of the 3 valid measurements.

##### Body Fat Mass and Muscle Mass

Body fat percentage (increments of 0.1%) and muscle mass (weight of muscle in the body) will be assessed using the Tanita InnerScan Body Composition Monitor (BC-545N, Tanita Corporation). The device uses Bioelectrical Impedance Analysis to determine body fat percentage and muscle mass. A total of 3 measurements will be recorded, and the average value will be recorded.

##### Blood Pressure and Pulse Rate

Blood pressure and heart rate will be measured using the Omron Auto Blood Pressure Monitor (HEM7121, Omron Healthcare). Participants will remain seated and relaxed, with 3 readings taken at least 3 minutes apart. The following parameters will be recorded: (1) systolic blood pressure, (2) diastolic blood pressure, and (3) heart rate (pulse, bpm). If the difference between the 3 systolic blood pressure or diastolic blood pressure readings exceeds 10 mm Hg or the heart rate varies by more than 10 bpm, additional measurements will be taken until 3 consecutive readings fall within these thresholds. The final recorded values will be the average of the last 2 valid measurements as the first readings of blood pressure and pulse rate are often less reliable due to initial participant adjustment.

##### Glucose and Lipids Screening Test

A nonfasting finger-prick blood test will be conducted using the CardioChek PA Analyzer, which provides the results within 2 minutes. The following biomarkers will be measured and recorded to the nearest mmol/L: (1) blood glucose, (2) total cholesterol, and (3) high-density lipoprotein. Each participant will undergo a single measurement.

##### Grip Strength

Grip strength (kg) will be assessed using a portable hand dynamometer, a validated measure of overall physical capability [66]. Participants will be instructed to grip the dynamometer as tightly as possible. A total of 3 trials will be conducted for each hand, and the maximum will be recorded for both the left and right hands [67].

##### Skin Carotenoid

Skin carotenoid concentration in the fingertip fat pad will be assessed using noninvasive reflectance spectroscopy with the Veggie Meter (Longevity Link). This method provides an objective and reliable biomarker for fruit and vegetable consumption, supported by previous studies [68-70]. The device functions by gently compressing the finger, temporarily displacing blood from the measurement area. It then emits light, analyzes the reflected light spectrum, and calculates a carotenoid score within a few minutes, which serves as a biomarker for dietary intake [70]. To ensure accuracy, measurements will be made using a multimeasurement mode, with 3 sequential readings within 10% of each other averaged over a 45-second period.

### Self-Reported Questionnaires

#### Psychological Well-Being

Psychological well-being will be assessed using the following scales:

1. The Kessler Psychological Distress Scale: The Kessler Psychological Distress Scale is a widely used measure of nonspecific psychological distress [71]. It has been validated across multiple settings and diverse populations, including in New Zealand [9,72].
2. Generalized Anxiety Disorder 7-item Scale: Participants will be assessed for symptoms of anxiety using the Generalized Anxiety Disorder 7-item scale [73], a self-report screening tool that has been validated for use with both adults and adolescents in the general population across many different cultures, demonstrating sound psychometric properties [74].
3. Beck Depression Inventory-Revised: Depressive symptoms will be measured using the Beck Depression Inventory-revised [75], a widely used screening tool with strong psychometric properties for assessing depression severity in adults and adolescents [76].
4. Adult Self-Report Behavior Checklist: Externalizing problem behaviors will be assessed using the aggression, rule-breaking, and intrusive behavior subscales of this checklist [77]. It assesses behavioral and emotional problems in adults aged 18‐59 over the past 6 months and has well-established psychometric validity [78].
5. World Health Organization-5 Well-Being Index: This 5-item questionnaire measures mental well-being and positive psychological functioning. Substantial empirical evidence supports its validity, reliability, and cross-cultural applicability [79,80].
6. World Health Organization Quality of Life-Brief Version: This 26-item instrument assesses quality of life across 4 domains: physical, psychological, social, and environmental. It has been extensively validated for reliability and cross-cultural relevance [81].
7. Pacific Identity and Wellbeing Scale-Revised: It is developed by a Cook Islands Māori researcher, this tool evaluates the influence of culture and identity on health outcomes [82]. The scale consists of 6 subscales: “Perceived Familial Wellbeing,” “Perceived Societal Wellbeing,” “Group Membership Evaluation,” “Pacific Connectedness and Belonging,” “Religious Centrality and Embeddedness,” and “Cultural Efficacy.” Cultural identity and engagement will be measured with the latter 5 subscales. Family functioning will be measured with the first subscale, “Perceived Familial Wellbeing.”. The Pacific Identity and Wellbeing Scale-Revised has demonstrated strong internal reliability, construct validity, and applicability across the 4 major Pacific ethnic groups [82,83].
8. Systemic Clinical Outcome and Routine Evaluation: This 15-item questionnaire assesses family functioning across 3 dimensions: strengths, difficulties, and communication [84]. Systemic Clinical Outcome and Routine Evaluation has demonstrated good internal and test-retest reliability and criterion validity [85].
9. Cognitive Emotion Regulation Questionnaire-Short: The Cognitive Emotion Regulation Questionnaire-Short assesses cognitive strategies individuals use to regulate emotions in response to stressful or threatening life events [86]. It consists of 18 items across 9 subscales: self-blame, other-blame, rumination, catastrophizing, positive refocusing, planning, positive reappraisal, putting into perspective, and acceptance. Higher subscale scores indicate a greater tendency to use the corresponding cognitive emotion regulation strategy [86].
10. Nature Relatedness-6: This scale measures an individual’s subjective connectedness to nature, with higher scores indicating stronger connection to the natural environment [87]. Prior research demonstrates that greater nature relatedness correlates with psychological well-being [88], reduced perceived stress [89], and lower anxiety levels [90], supporting its use in examining psychological well-being among Pacific young adults.
11. The Normalized Difference Vegetation Index: Vegetation density around participants’ residential neighborhoods will be quantified using Normalized Difference Vegetation Index methodology [91]. Following geocoding of participants’ home addresses, Normalized Difference Vegetation Index values will be derived through spectral analysis comparing near-infrared and visible light reflectance in order to examine the link between residential green space and psychological well-being. In addition to vegetation density, residential distance to nearest green spaces will also be analyzed. Studies have linked greener spaces to lower depression, anxiety, and stress levels [92,93].

#### Nutritional and Metabolic Well-Being

To assess nutritional and metabolic well-being, the following questionnaires will be used:

1. Food Frequency Questionnaire: Dietary patterns will be assessed using a validated 57-item short version of the Food Frequency Questionnaire [94]. This short version has been validated for use with New Zealand subpopulation groups. It captures data on food consumption (eg, bread, potatoes, and protein sources) to estimate nutrient intake [94].
2. Household Food Security: Household food security will be assessed using an 8-item questionnaire designed to measure access to nutritionally adequate and safe food. This tool has been widely used in various phases of the New Zealand Health Survey [95].
3. International Physical Activity Questionnaire-Short Form: This questionnaire measures physical activity levels, including types and intensity of activity and sedentary behavior, to estimate total physical activity in days per week and minutes and hours per day [96].
4. Sedentary Behavior Questionnaire: Sedentary behaviors will be evaluated using the Sedentary Behavior Questionnaire, a validated tool assessing habitual sedentariness across occupational, transportation, household, and leisure-time domains. Given the link between sedentary behavior and cardiovascular risks, the Sedentary Behavior Questionnaire provides a comprehensive measure with strong psychometric properties [97].

#### Economic Well-Being

The following items will be used to assess economic well-being:

1. Tertiary course completion: This will be measured by tracking all courses attempted at universities, institutes of technology and polytechnics, wānanga (publicly owned tertiary institutions or Māori universities that offer education rooted in Māori cultural values), industry training organizations, and private training establishments.
2. Employment status: Employment status will be categorized as unemployed, employed part-time, employed full-time, or not in the labor force, based on standard questions from Statistics New Zealand’s Household Labour Force Survey [98].
3. Occupation: Occupation will be assessed using standard questions from the New Zealand Census and coded according to the Australian and New Zealand Standard Classification of Occupations, which ranks jobs based on skill level and specialization [99].
4. Industry of employment: This will be identified using standard questions from Statistics New Zealand’s Household Labor Force Survey and coded according to the Australian and New Zealand Standard Industrial Classification [99].

In addition to these core measures, there will be supplementary questionnaires to examine key factors influencing economic outcomes. These questionnaires will explore (1) reasons for tertiary course incompletion, (2) reasons for unemployment or being out of the labor force, and (3) factors influencing occupational and industry choices. The development of these items will be informed by validated measures from the Household Labor Force Survey [98], as well as findings from the Graduate Longitudinal Study New Zealand, which identifies factors that facilitate or hinder qualification completion among tertiary students [100,101].

#### Sociodemographic Questions

Additional sociodemographic and contextual information will be collected, including gender, ethnicity, spoken languages, faith or religion, sexual orientation, household size, relationship status, and parental status.

### Data Management

All digital data will be securely stored on REDCap (Research Electronic Data Capture; Vanderbilt University), a secure, web-based software platform [102,103], with backup copies maintained on the AUT-supported Microsoft Teams platform. For participants who complete paper forms, either by choice or due to internet connectivity issues, these forms will be securely stored in lockable filing cabinets at AUT South Campus. Access to this storage area is restricted by swipe card and limited to authorized researchers directly involved in the study.

Access to the database of participants’ contact details is restricted to researchers and field interviewers employed on the PIF Study. Permissions to access this data are managed by the PIF Study Director. For external collaborators, such as named investigators from other institutions involved in PIF Study projects, access to research data is facilitated through a dedicated, project-specific Microsoft Teams site. Only researchers directly associated with the specific project are granted access to this site. All PIF:ATP data will be securely retained for 10 years following the conclusion of the PIF Study, after which it will be permanently destroyed.

### Data Analysis: Statistical Considerations

Reporting of all findings for each study will comply with the Strengthening the Reporting of Observational studies in Epidemiology (STROBE) guidelines [104]. A completed STROBE checklist is provided as Checklist 1 to document the reporting standards for each study within the PIF:ATP program. All analyses will be performed using contemporary specialist software, such as Stata SE version 18.0 (StataCorp). Results will be interpreted based on both statistical significance (2-sided *α*=.05) and effect sizes. Following a description of participant flow (recruitment and attrition) and characteristics, crude frequencies of primary exposure and outcome variables across measurement waves will be reported.

Important social determinants of health such as family size, household income, neighborhood socioeconomic deprivation measured contemporaneously with psychological, nutritional and metabolic, and economic outcomes will be included in the analyses. Where appropriate, analyses will also incorporate antecedent data collected over the cohort’s life course including childhood family circumstances from birth to adolescence. All findings will be contextualized within the broader historical, social, political, and economic circumstances in which they occurred. This approach ensures that structural determinants, including migration, systemic inequities, housing, and policy changes, are considered.

Apposite longitudinal models will be developed for each study, taking into account various considerations such as the distribution of outcome variables (eg, binary when thresholds are used for Kessler Psychological Distress Scale and Generalized Anxiety Disorder 7-item scale). Distributional assumptions for continuous variables will be checked, and transformations will be applied where necessary (eg, logarithmic transformations for BMI). Measurement wave time will also be included in all longitudinal analysis. Patterns over time will be investigated using degree-2 fractional polynomial regression models [105], or indicator variables when the number of measurement waves is relatively small. Group-based multitrajectory modeling will also be used when multiple outcomes are simultaneously considered (eg, investigating dietary patterns) [106].

Multitrajectory modeling identifies latent clusters of individuals following similar trajectories over time across multiple outcome indicators. Although relatively recent, this technique has been rapidly adopted and applied to longitudinal research of psychological and psychiatric outcomes in adolescents and young adults [107]. All longitudinal analysis will accommodate within-person correlations, modeled using unstructured or exchangeable matrices, and Huber-White robust sandwich estimators of variance will be used to provide insurance against model specifications. Generalized estimating equations will be used for population-averaged panel-data models, while multilevel mixed effects models will be used for models containing both fixed effects and random effects and hierarchical structures. Both these statistical approaches have been successfully used in many previous PIF Study analyses and publications [25,40,108].

To assess model predictive ability, a key factor in establishing causality, Lasso methods will be used [109]. Unlike techniques that evaluate models in the hold-out sample, Lasso helps to avoid overfitting by minimizing an estimate of the out-of-sample prediction error. K-fold cross-validation will also be used as a check, and the 10-fold cross-validated area under the receiver operating characteristic curve (AUC) will be used for binary variables. In k-fold cross-validation, the dataset is randomly partitioned into k approximately equal subsamples (or folds). At each iteration, one fold is retained as the validation data for testing the model and estimating the AUC, while the remaining k-1 folds are used as training data for model estimation. This process is repeated k times, with each fold used once as validation data [110]. K-fold cross-validation and Lasso avoid the optimistic estimates of predictive performance that can occur when the same data are used for both model specification and predictive assessment. Patterns of attrition will be assessed using binary Generalized Estimating Equation models. Following complete case analyses, sensitivity analyses will be conducted, where data from participants who attrite or have missing values will be imputed. As in previous PIF Study analyses and publications [25,111,112], multiple imputation (MI) methods will be used, and the data will be reanalyzed.

### Pacific Data Governance

The development of Pacific data principles by the Pacific Data Sovereignty Network has provided culturally centered frameworks for use when making decisions about how to approach Pacific data. The Pacific Data Sovereignty Network supports the notion that Pacific data should be subject to Pacific governance, and Pacific organizations should be able to access Pacific data to support their development aspirations or work that benefits Pacific families and communities [113].

With these principles in mind, the PIF:ATP team has put in place 2 advisory groups, an expert science group and an expert community liaison group, to provide Pacific guidance, cultural support, and scientific feedback from a Pacific perspective. All members are of Pacific descent and have long-standing careers and relationships working and interacting with Pacific families and communities. Alongside the PIF:ATP research investigators (of whom most are of Pacific descent), this collaborative network ensures several layers of Pacific governance and oversight are used to deliver on the principles of Pacific data sovereignty. Moreover, several members of the research team are part of the Pacific Data Sovereignty Network Advisory group and can bring this expertise and network of support and collaboration to the study if necessary.

Information from the study is also regularly disseminated back to families and communities through various channels including community fono (meetings), summary booklets, Pacific language videos, social media platforms, and traditional academic outputs for those interested, such as journal articles and conference presentations. Many of these forums act as a means of “sharing” where data are presented and audiences are able to provide feedback and interact with researchers. This feedback process has proven beneficial in previous measurement waves and provided guidance on priority issues for Pacific families, which we have been able to explore and examine in subsequent data collection waves.

### Ethical Considerations

This study received funding in October 2024 (reference number: Health Research Council of New Zealand 24/623) and was granted ethical approval in April 2025 by the Health and Disability Ethics Committee (reference number: 2025 EXP 22302) and in May 2025 by the Auckland University of Technology Ethics Committee (reference number: 25/165). Participants will receive an information sheet and consent form via email or post, depending on their preference. After reviewing the materials and confirming willingness to participate, participants will sign the consent form electronically via REDCap [102,103] before any data collection begins. Participation is entirely voluntary, and participants may withdraw at any time without consequence. As a token of appreciation, participants will receive a NZ $100 (~US $57.77) mea’alofa (gift) voucher for their valuable contribution. Those opting to complete their assessment at the AUT laboratory will also receive a NZ $30 (~US $17.33) petrol voucher to help cover travel expenses to and from campus.

## Results

Data collection is scheduled to commence in June 2025 and is anticipated to conclude by December 2026. Based on the attrition rates observed in previous waves, we aim to recruit approximately 750 cohort members. Data analysis will begin in January 2027, with the first results expected to be published December 2027 onward.

## Discussion

### Principal Findings

The PIF:ATP is the 25-year phase of the only prospective study focusing specifically on Pacific peoples. It will provide valuable insights into the long-term interrelations among psychological, nutritional and metabolic, and economic well-being in Pacific young adults. The findings will illuminate how mental distress, dietary patterns, and economic situation interact over time. In addition, the study will identify both cross-sectional and longitudinal risk and protective factors associated with mental health, nutrition, and economic well-being. By identifying these factors, this research aims to inform future interventions and policies designed to improve the mental health, dietary behaviors, and economic outcomes of Pacific young adults.

### Strengths and Limitations

The unique PIF:ATP program is well positioned to identify health and well-being trajectories from birth to early adulthood, determine relationships between early and later life events, and make causal inferences and identify potential interventions. Nevertheless, the major limitation of the study is the potential impact of sample attrition on the number and profile of participants. While we will check the patterns of attrition as in previous PIF publications [8,13,114], it remains a concern as differential attrition can introduce bias. If certain groups of participants (eg, those with poorer health or who have migrated to Australia) are more likely to not be measured, it may limit the generalizability of our findings. To account for this, we will evaluate the impact of attrition on our results and ensure that our conclusions remain robust.

Given that the previous wave experienced lower participation rates, which highlighted the challenges of locating and contacting participants, we will implement targeted strategies to enhance retention and re-engagement. These include prioritizing the recruitment of field researchers with strong connections to Pacific communities in Auckland. Their cultural understanding and established networks will be critical for encouraging participation. Additionally, we will use social media engagement strategies to increase visibility and encourage continued involvement. We will also extend the data collection period to 18 months, compared with the previous standard 12-month timeline, to reach more participants.

### Conclusions

The PIF:ATP research program will measure outcomes in psychological well-being, nutritional and metabolic well-being, and economic well-being among 25-year-old Pacific young adults. By addressing a significant gap in knowledge, this study will build a strong evidence base to inform health and well-being strategies and make services and health promotion activities more accessible, acceptable, and effective for Pacific young adults. The PIF:ATP program is uniquely positioned to leverage its comprehensive dataset spanning 25 years of the cohort’s life course, addressing key issues identified by Pacific communities and advancing knowledge on the well-being of Pacific young people.

## Supplementary material

10.2196/77460 Checklist 1 STROBE checklist.

## Abbreviations

ATP: Ala mo Tupulaga Pasifika
AUT: Auckland University of Technology
GAD-7: Generalized Anxiety Disorder 7-item scale
NCD: noncommunicable diseases
PIF: Pacific Islands Families study
REDCap: Research Electronic Data Capture
STROBE: Strengthening the Reporting of Observational studies in Epidemiology
